# Spectrophotometric Determination of Diazepam in Pure form, Tablets and Ampoules

**Published:** 2007-03

**Authors:** W. F. El-Hawary, Y. M. Issa, A. Talat

**Affiliations:** *Chemistry Department, Faculty of Science, Cairo University, Giza, Egypt*

**Keywords:** diazepam, 2,4-dinitrobenzoic acid, 3,5-dinitrobenzoic acid, picric acid, spectrophotometric determination

## Abstract

The interaction of diazepam with picric acid (I), 3,5-dinitrobenzoic acid (II) and 2,4-dinitrobenzoic acid (III) was found to be useful for its spectrophotometric determination. The quantitation was carried out at 475, 500, and 500 nm for the reaction with (I), (II) and (III), respectively. The effect of several variables on the coloring process was studied. The proposed methods have been applied successfully for the determination of diazepam in pure samples and in its pharmaceutical preparations with good accuracy and precision. The results were compared to those obtained by the pharmacopoeial methods. The linear ranges for obedience of Beer’s law are up to 85.6, 180.2, and 128.6 µg/ml, Ringbom ranges are 10.0-79.0, 15.2-177.8, 17.0-83.0 µg/ml, and RSD 0.048, 0.028, and 0.026% for reaction of diazepam with I, II, and III, respectively.

## INTRODUCTION

Diazepam (C_16_H_13_ClN_2_O) [7-chloro-1,3-dihydro-1-methyl-5-phenyl-2H-1,4-benzodiazpein], M. Wt. 284.75 [CAS (439-14-5)] is an important compound widely used therapeutically because of its relaxant, sedative, hypnotic and anticonvulsant properties.

Several analytical procedures have been adapted for the assay of diazepam. They include non-aqueous titratimetry (1, 2), ultraviolet spectrophotometry (3-5), visible spectrophotometry (6-10), second order-derivative spectrophotometry (11, 12), fluorimetry (13, 14), high performance liquid chromatography HPLC (15-17), gas chromatography (18, 19), thin layer chromatography (20), polarography (21, 22), potentiometry (23, 24) and infrared assay (25). The official methods involve a non-aqueous titration of diazepam by perchloric acid in acetic anhydride medium using Nile blue as indicator (2) and HPLC (17).

Most of the old colorimetric methods involve hydrolysis of the benzodiazepine moiety to benzophenones, and thus lack specificity since this is the usual degradation pathway of benzodiazepines, and other needs solvent extraction before measurements. Therefore, a simple spectro-photometric method for determination of diazepam is needed. This is fulfilled in the present investigation by the application of Zimmermann reaction to the active methylene group adjacent to a carbonyl group in diazepam to produce highly absorbing σ-complexes (26) upon reaction with picric acid (I), 3,5-dinitrobenzoic acid (II) and 2,4-dinitrobenzoic acid (III), respectively. The present study describes the spectrophotometric determination of diazepam in pure samples and in its pharmaceutical preparations.

## EXPERIMENTAL

### Apparatus

Perkin Elmer Spectrophotometer model Lambda 1, Hanna instrument coductometer model HI8819N and Hanna pH meter model HI3313N were used for measuring absorbance, conductance and pH values, respectively.

### Materials

Diazepam [7-chloro-1,3-dihydro-1-methyl-5-phenyl-2H-1,4-benzo-diazpein] (M. Wt.=284.75) was obtained from Memephis Co., Egypt and its purity was determined by the U.S. pharmacopoeial XX method (1). The pharmaceutical preparations (Farcozepam®, tablets 2 mg/tablet and Valepam® ampoules, 10 mg/ampoule) were purchased from the local market (Pharco Co., Egypt). All reagents were of analytical pure grade. They include sodium hydroxide, ethyl alcohol (99%), perchloric acid, acetic anhydride, Nile blue indicator, picric acid (I), 3,5-dinitrobenzoic acid (II) and 2,4-dinitrobenzoic acid (III).

### Stock solutions

5 × 10^-2^ M alcoholic solution of diazepam was prepared and standardized by titration in acetic anhydride medium with HClO_4_ dissolved in glacial acetic acid, using Nile blue hydrochloride as indicator (2). Further dilution were made to 3 × 10^-3^ M, 1.5 × 10^-2^ M and 5 × 10^-3^ M. Alcoholic solutions of the electron acceptors were prepared at concentrations of 3 × 10^-3^ M of (I), 1.5 × 10^-2^ M of (II) and 5 × 10^-3^ M of (III). The pH of the medium was adjusted using 5,7 and 10 M sodium hydroxide solutions.

### Procedure

A solution containing 10.0-79.0 (in case of reaction with I), 15.2-177.8 (in case of reaction with II) or 17.0-83.0 µg/ml (in case of reaction with III) of diazepam was transferred into 10 ml measuring flask. 0.7 ml of 3.0 × 10^-3^ M of I, 2 ml of 1.5 × 10^-2^ M of II or 0.6 ml of 5 × 10^-3^ M of III, respectively was then added, followed by the appropriate amount of 5, 7 or 10 M sodium hydroxide to give a final concentrations of 0.5, 2.5 or 4.0 M, respectively. The volume was then completed up to 10 ml with alcohol, shaked well and left for 50, 60 or 60 minutes, respectively, at room temperature for full color development. The formed complexes remained stable for 1 day, 90 and 90 minutes for I, II and III, respectively. The absorbances were then measured at 475, 500 and 500 nm, respectively.

### Application to pharmaceutical preparations

**Farcozepam^®^ tablets.** The developed procedure was applied for the determination of diazepam in some dosage forms without prior separation. Thirty tablets of (Farcozepam 2 mg) were weighed accurately and powdered in an agate mortar. An amount corresponding to 20 mg of diazepam was transferred to a flask containing 30 ml of alcohol and the suspension was shaked with a mechanical shaker for 30 minutes, followed by treating for 1 minute in a bath subjected to the action of ultrasonic waves then filtered, transferred to 50 ml measuring flask and diluted to the mark with ethyl alcohol. An aliquot was transferred to 10 ml measuring flask and treated as previously described. The concentrations of the drug were obtained from the calibration curve of diazepam and the recoveries, applying the new method, were calculated.

**Valepam^®^ ampoules.** Solution of Valepam^®^ ampoule (10 mg/ampoule) was prepared by mixing the contents of 5 ampoules. Then 1.5 ml of this solution was diluted to 25 ml with ethyl alcohol in a measuring flask. An aliquot was transferred to 10 ml measuring flask and treated as previously described. The concentrations of the drug were obtained from the calibration curve of diazepam and the recoveries, applying the new method, were calculated.

## RESULTS AND DISCUSSION

### Formation of the complexes and determination of their stability constants

The reaction of picric acid (I), 3,5-dinitrobenzoic acid (II) and 2,4-dinitrobenzoic acid (III) with active methylene compounds in alkaline medium is known to proceed via the formation of σ-complexes (26). The complex is called Meisenheimer complex and the reaction is called Janovsky reaction. In the presence of excess I, II and III, the complex is oxidized to a coloured anion while the reagents are reduced to 2-amino-4, 6-dinitrophenol, 3-amino-5-nitro-benzoic acid and 2-amino-4-nitro-benzoic acid, respectively, under Zimmermann conditions (27).

Diazepam was found to yield intensely red coloured products in case of reaction with I, II and III, whose maximum absorbances were found at 475, 500 and 500 nm, respectively, most probably due to formation of σ-complexes between diazepam and I, II and III.

The ratio of (diazepam:reagent) in the formed complexes was determined by using the molar ratio method (28) and conductimetric titration (29). Application of molar ratio method indicates the formation of 1:1 in case of I, 1:1 and 1:2 in case of II, and 1:1 in case of III (diazepam:reagent) complexes, respectively. Application of conductimetric titration indicates the formation of 1:1 in case of I, 1:1 and 1:2 in case of II and 1:1 in case of III (diazepam: reagent) complexes, respectively.

The stability constants of the complexes formed between diazepam and I, II or III, were calculated using Harvey and Manning method (30). The stability constants, β_n_, of the formed complexes were calculated applying molar ratio method by the aid of the following equation:

$\beta_{n}=\frac{A/A_{m}}{{\left(1-A/A_{m}\right)}^{n+1}{\left(C_{D}\right)}^{n}n^{2}}$

where A is the absorbance at the drug concentration C_D_; A_m_ is the absorbance at full color development; N is the stoichiometric ratio of the complex; C_D_ is the concentration of drug.

The values of stability constants of the formed complexes are depicted in Table 1. It was found that the sequence of increasing stability of the complexes is I<III<II.

**Table 1:** Stability constants of the complexes of diazepam using the molar ratio method (MRM)

Complex	Method	Mole ratio	K_n_	β

Picric acid	MRM	1:1	6.67 × 10^3^	6.67 × 10^3^
3,5-Dinitrobenzoic acid		1:1	2.09 × 10^4^	2.65 × 10^11^
	MRM	1:2	1.27 × 10^7^	
2,4-Dinitrobenzoic acid	MRM	1:1	14284	1.43 × 10^4^

MRM, Molar ratio method; K_n_, Stability constant; β, Overall formation constant.

### Determination of diazepam

The formation of the above mentioned complexes was utilized for the spectrophotometric determination of diazepam in pure form, by measuring the absorbances of the formed complexes with I, II and III at 475, 500 and 500 nm, respectively. Table 2 summarizes the different parameters of this determinations, i.e. the wavelength of maximum absorption (λ_max_), molar absorptivity (ε), specific absorptivity (a), Sandell's sensitivity ($), range of obedience of Beer's law, Ringbom range and the statisticals of the calibration curve. The results shown in Table 2 reveal that Beer's law is obeyed up to 85.6, 180.2 and 128.6 µg/ml of diazepam in case of determination using I, II and III, respectively; with detection limits of 10.0, 15.2 and 17.0 µg/ml, respectively.

**Table 2:** Wavelength for maximum absorption, molar absorptivity, specific absorptivity, Sandell’s sensitivity, Ringbom range and statistical studies in case of diazepam complexes

	Picric acid	3,5-dinitrobenzoic acid	2,4-dinitrobenzoic acid

λ_max_nm	475	500	500
ε	2.89 × 10^3^	1.03 × 10^3^	4.30 × 10^3^
a	10.14	3.63	15.00
I	-0.02	0.05	-0.20
r	1.00	0.99	0.99
S	4.79 ×10^-3^	8.03 ×10^-2^	7.20 ×10^-2^
$ ×10^5^	9.86	27.55	6.67
Conc µg/ml*	85.6	180.6	128.6
Ringbom range µg/ml	10.0-79.0	15.2-177.8	17.0-83.0

λ_max_, Wavelength for maximum absorption (nm); ε, Molar absorptivity l mol^-1^ cm^-1^; a, Specific absorptivity l g^-1^ cm^-1^; I, Intercept of calibration curve; r, Correlation coefficient of the calibration curve; S, Standard deviation of the calibration curve; $, Sandell’s sensitivity µg cm^-2^.

* Obedience of Beer’s law up to this concentration.

The applicability of the proposed methods was tested, also, for the determination of diazepam in pharmaceutical preparations. It was found that the proposed methods can be applied successfully for the determination of diazepam both in pure form and in pharmaceutical preparations. Table 3 summarizes the results of such determinations. The mg taken of the drug [as determined by the official method (2)] is shown versus the mg found by the proposed methods.The good recoveries obtained (98.9-101.3%) indicates the accuracy of these methods. Also, the precisions of the methods were tested by calculating the relative standard deviations for different determinations. The results shown in Table 3 indicate low values of the relative standard deviation, which taken as an evidence for the precision of the present methods.

**Table 3:** Determination of diazepam in pure solution, tablets and ampoules

	Pure solution					Tablet solution					Ampoule solution				
	Taken^a^ (mg)	Found (mg)	R (%)	RSD	F-Value^b^	Taken^a^ (mg)	Found (mg)	R (%)	RSD	F-Value^b^	Taken^a^ (mg)	Found (mg)	R (%)	RSD	F-Value^b^

Picric acid	0.10	0.100	100.00	0.048	3.10	0.15	0.150	100.00	0.013	3.13	0.20	0.200	100.00	0.032	3.15
	0.30	0.297	99.00			0.30	0.297	99.00			0.30	0.297	99.00		
	0.40	0.400	100.00			0.45	0.452	100.44			0.40	0.404	101.00		
	0.45	0.446	99.00			0.50	0.500	100.00			0.50	0.495	99.00		
	0.50	0.490	99.00			0.60	0594	99.10			0.60	0.600	100.00			
	0.60	0.600	100.00			0.70	0.693	99.00			0.70	0.700	100.00		
	0.75	0.742	99.00												
3,5-Dinitrobenzoic acid	0.20	0.197	98.80	0.028	4.09	0.20	0.197	98.80	0.022	4.75	0.20	0.197	98.80	0.019	4.47
	0.40	0.398	99.60			0.40	0.398	99.60			0.40	0.398	99.60		
	0.60	0.597	99.50			0.60	0.592	99.00			0.60	0.592	99.00		
	0.90	0.906	100.70			0.90	0.906	100.70			0.90	0.906	100.70		
	1.10	1.105	100.20			1.10	1.102	100.20			1.00	0.996	99.60		
	1.20	1.215	101.30			1.70	1.717	101.00			1.10	1.102	100.20		
	1.70	1.717	101.00								1.70	1.717	101.00		
2,4-Dinitrobenzoic acid	0.30	0.297	99.00	0.026	2.06	0.20	0.200	100.00	0.031	1.80	0.18	0.180	100.00	0.015	1.72
	0.40	0.400	100.00			0.45	0.450	100.00			0.30	0.297	99.00		
	0.60	0.594	99.00			0.70	0.693	99.00			0.40	0.397	99.25		
	0.80	0.796	99.50			1.07	1.062	99.20			0.68	0.680			
	1.00	1.000	100.00			1.34	1.327	99.00			0.78	0.780	100.00		
	1.20	1.181	98.40								0.94	0.940	100.00		
											1.00	1.012	101.20		
											1.10	1.086	98.70		
											1.34	1.327	99.00		

Tabulated F-value (99% confidence level) at ν_1_ = 6 (official method) and ν_2_= 5 (new method) is 5.95, where ν is the degree of freedom. R, Recovery; RSD, Relative standard deviation.

a mg taken as determined by the official method;

b F-value with respect to official method.

In order to assess the accuracy and precision of the present methods, the mean values obtained by the proposed methods were compared with each other using t-test,and the variances were compared with those of the official one (2) using F-test. The obtained t-values range from 0.48 to 1.92, which are lower than the tabulated value at 99% confidence level and 12 degrees of freedom (3.06). The obtained F-values range from 1.72 to 4.75, which are lower than the tabulated value at 99% confidence level and 6, 5 degrees of freedom for the official and proposed methods, respectively (5.95). This means that there is no significant difference in accuracy of the proposed methods. Also, the proposed methods are of comparable precision with the official ones at 99% confidence level as shown by the calculated "t" and "F" values shown in Tables 3 and 4.

**Table 4:** Comparison between different methods used for determination of diazepam using t- test

	Picric acid	3,5-dinitrobenzoic acid	2,4-dinitrobenzoic acid	Official method (1, 2)

Picric acid	--------	--------	--------	0.652
3,5-dinitrobenzoic acid	0.866	--------	--------	0.477
2,4-dinitrobenzoic acid	0.576	1.746	--------	1.921

Theoretical value for t-value at 12 degree of freedom and at 99% confidence level is 3.06.

The pharmacopoeial methods (2, 17) for determination of diazepam in the raw material, tablets, and injection depends on non-aqueous titration with 0.1 N perchloric acid in acetic anhydride medium, using 1% solution of Nile blue hydrochloride in glacial acetic acid as indicator to yellowish-green end point (2) or HPLC (17). The minimum quantities determined by these methods are 1 mg/ml or 10 mg/ml in case of USP (17) or British pharmacopoeia (2), respectively; as well as the technique needs certain precautions to keep anhydrous medium. The proposed methods are used for the determination of much lower concentration (10 µg/ml), as well as it has a high reproducibility.

The effect of interference of different cations and anions on the absorbances of the formed complex was studied and it was found that in case of complexes of diazepam with picric acid, 3,5-dinitrobenzoic acid and 2,4-dinitrobenzoic acid, up to 20 folds of Na^+^, Mg^2+^, K^+^, Zn^2+^ Pb^2+^, PO_4_^3-^, Cl^-^, I^-^, Br^-^, S_2_O_3_^2-^, SO_4_^2-^, oxalate, citrate, tartrate, salicylate, acetate, nitroprusside, gluconate, pyroborate, hydrogen tartrate, metvanadate, sucrose, lactose, dextrose, glutamine, glycine and L-aspragine do not interfere. On the other hand, Ba^2+^, Ca^2+^, Fe^2+^, Fe^3+^, Hg^2+^, CN^-^, NO_2_^-^, SCN^-^, MnO_4_^-^, Cr_2_O_7_^2-^, and EDTA interfere.

Various spectrophotometric methods have been used for determination of diazepam. The method proposed by Sadeghi (6) is based on the reaction of diazepam with bromocresol green at pH 3.5, extracting the colored product into chloroform and measuring the absorbance of chloroform layer at 410 nm. Although this method is sensitive (Beer’s law is obeyed within the range 2-60 µg/ml), but it needs tedious and time consuming extraction procedure. The methods proposed by Popovici depends on the reaction of diazepam with picric in benzene (7) or chloroform (8) medium to form a colored product which is measured at 400 nm. Beer’s law is obeyed within the range 20-300 µg/ml. The minimum quantity determined by this method is 20 µg/ml, as well as the benzene and chloroform solvents are expensive and have carcinogenic effect. Another spectrophotometric technique (9) is based on extraction of diazepam from hydrochloric acid medium into dichloromethane (CH_2_Cl_2_) as a colored ion pair complex with orange II. Beer’s law is obeyed from 0.6-10 µg/ml, the molar absorptivity (ε) value is 1.15 × 10^4^. This method is very sensitive, but in needs, also, prior extraction with organic solvent. The spectrophotometric method based on extraction of diazepam from aqueous solution at pH 1.2 into chloroform as a colored complex with Alizarin violet 3B or Alizarin brilliant violet R which is measured at 560 nm (10), has also the disadvantage of prior extraction with a harmful organic solvent. Beer’s law was obeyed from 4-16 µg/ml.

Comparing the proposed methods with the published ones reveals that the new methods are simple, need no prior separation steps and can be applied for determination of very low concentrations (10 µg/ml) with corresponding coefficient of variation ranges from 2.6-4.8% (n=7). Also, the reagents used are common and available. In conclusion, the new methods are comparable in accuracy and precision with the published ones.

## CONCLUSION

In conclusion, the proposed procedures are simple, inexpensive, and more sensitive than the official method. The developed procedures can be applied to the determination of diazepam in some dosage forms without prior separation. The developed procedure, being simple and rapid, can be recommended for routine analysis in drug quality control laboratories. Recovery experiments were carried out for the drug in its respective formulation. The excellent recoveries indicate the absence of interference from frequently encountered excipients or additives.
